# Impact of professional, recreational and nonsinging on temporomandibular disorders - a comparative study based on a self-assessment questionnaire

**DOI:** 10.1186/s13005-024-00419-z

**Published:** 2024-03-21

**Authors:** Maja Wollenburg, Anne Wolowski

**Affiliations:** Department of Prosthodontics, University Hospital Münster, Albert-Schweitzer-Campus 1/W30, Münster, 48149 Germany

**Keywords:** Temporomandibular disorders, TMDs-prevalence, Singing, Vocalists, Work-related craniomandibular disorders

## Abstract

**Background:**

This study investigates the relationship between professional and recreational singing on temporomandibular disorders (TMDs) in women compared to a nonsinging control group.

**Methods:**

A total of 288 female subjects between the ages of 18 and 45 participated in the self-assessment questionnaire including demographic data, as well as questions on vocal practice and TMDs symptoms. Depending on the singing time per week, the (non)vocalists were assigned to the groups professional (*n* = 96), recreational (*n* = 96) and nonsingers (*n* = 96).

**Results:**

The TMDs prevalence in professional singers (42%) was higher than that in recreational singers (31%) and noticeably higher than that in nonsingers (25%). The Fisher-Freeman-Halton exact test showed that the differences between the groups were not noticeable (*p* = .053) but could be formulated as tendencies. The professionals suffered much more from restricted jaw movement (*p* = .004; OR = 2.718; 95% CI = 1.409–5.242), temporomandibular joint sounds (*p* < .009; OR = 2.267; 95% CI = 1.264–4.064) and temporomandibular pain (*p* = .010; OR = 2.333; 95% CI = 1.264–4.308) than nonsingers.

**Conclusions:**

Singing might have an enhancing effect on the appearance of TMDs. In particular, professional singers suffered more from self-reported TMDs than recreational singers and nonsingers. In addition to the high level of physical workload if participating in professional singing, the psychosocial impact should be investigated more in further studies. No new treatment strategies resulted from this study, as the etiological significance of singing is still unclear. Knowledge about risk factors for multifactorial TMDs can help practitioners and patients prevent and treat TMDs.

## Background

The masticatory organ is strained during everyday activities such as speaking, laughing, eating, singing and many more. When this gets out of balance, we often speak of temporomandibular disorders (TMDs). TMDs is an umbrella term for orofacial problems that affect the temporomandibular joints, the masticatory muscles, bone components and surrounding soft tissue [1–3]. However, TMDs should not be interpreted and used as a diagnostic term but requires further diagnostics regarding the existing disorder or more often disorders [4]. The emphasis on the plural is important: many health disorders and accompanying medical conditions associated with TMDs, which are described in the literature with different prevalences of between 5 and 31% [5–7]. The sheer number of individual TMDs and the fact that disorders in the orofacial region can affect function, anatomical structures and/or physiology illustrates the versatility of TMDs and the need for individualised treatment for those affected [4, 8]. Most TMDs can be categorised as pain and/or dysfunction. Those affected can sometimes only perform jaw movements to a limited extent and/or suffer from persistent pain or other restrictive conditions. This can have a drastic negative impact on the quality of life of those affected [9–12] and their personal health - starting with sleep disorders [13], continuing with depression [14] and ending with suicidal intentions [15]. That is why it is more topical and important than ever to research risk factors to develop suitable individualised treatment strategies and to pursue prevention. The etiology of TMDs is very complex, interdisciplinary and not yet fully understood [16, 17]. Some biological factors - such as female gender, professional athletes, age - and psychosocial factors - such as poor stress management and competitive pressure - have been documented in the literature [9, 18–26]. Since athletes, for example, represent a risk group for TMDs [19, 27], it is reasonable to assume that this correlation could also be transferred to athletes in the orofacial region, such as singers.

According to the German Music Information Centre, at least 14 million people -, i.e., approximately one in six in Germany - make music in their free time or for professional purposes [28]. The proportion of singers is not given but will be similar. Nevertheless, this illustrates the size and importance of the potential risk group of singers. To consider the quantitative aspect of singing and the resulting strain on the masticatory organ, the female singers were divided into professional and recreational singers and analysed to determine whether there was an increased prevalence of TMDs in one of the cohorts compared to a control group. The aim of this descriptive cross-sectional study was therefore to search for further risk factors, namely the influence of professional, recreational and nonsinging on the masticatory organ.

Correlations between TMDs and singing have been studied for three decades, but the causal relationship between singing and the development of TMDs is not clearly established in the literature. Some authors have suggested that singing may be a predisposing factor for TMDs complaints. However, no study currently differentiates between professional and recreational singing.

Taddey described a connection between singing and the development of TMDs as early as 30 years ago [29]. Three singing patients experienced relief of TMDs symptoms by reducing their singing time. The main trigger for the development of TMDs in singers, as well as in trombone players, was quantitatively high exercise. He described a correlation that is supported by recent meta-analyses and reviews [30–32]. Since Taddey’s study design was not published, it remains unknown whether other factors, such as gender, professional or recreational practice and age, limited his results.

In 2007, Franco and Andrus confirmed that in professional singers, an incorrect singing technique and especially a quantitatively high, intensive strain can lead to an overload of the masticatory apparatus [33]. According to the authors, excessive singing in the sense of overstraining without sufficient recovery time - as in professional sports - was the main trigger for complaints in the head and neck region.

A review from 2017 related to musicians and singers stated that there is currently no scientific evidence for a significantly higher incidence of TMDs because of excessive muscle tension in the neck and larynx during singing [34]. For this review, the authors excluded Taddey’s publication due to the lack of information on the study design and denied a significant association between singing and TMDs complaints [34, 35]. According to the authors, slightly higher prevalences in classical singers were due to other confounding factors, such as age and gender [36]. Fifteen of 59 published articles were included in the review, of which only one publication considered singers: in 2011, a survey was conducted in Sao Paulo on 50 professional choral singers (29 women, 21 men) and 150 control subjects (87 women, 63 men) aged 18 to 63 years, focusing on existing physical complaints. The professional singers had less pain than the control group in various parts of the body but also around the jaw. The authors explained this by the fact that professional singers strengthen and relax their muscles through a routine of exercises [37, 38]. All singers pursued a profession in addition to singing. Thus, the “professional singers” classified according to Vaiano are part of the cohort of recreational singers defined in this study because they pursue another profession for a living and presumably perform singing as part of their leisure activities. Accordingly, they were listed as recreational singers in Table 1.

**Table 1:** Literature comparison based on the following criteria: number of cases (N), age restriction, gender distribution, study population (professional (P) and/or recreational (R) singers) and the research question (whether singing promotes TMDs)

		Literature comparison			
	N	Age	Gender	(R) and/or (P)	Singing → TMDs
Caetano et al.	33	18–75 y.	♂ + ♀	(R)	Not directly
Franco and Andrus	-	-	-	(P)	Yes
Grape et al.	16	n. s.	♂ + ♀	(R) + (P)	Not directly
Taddey	3	n. s.	♂ + ♀	-	Yes
Vaiano et al.	200	18–63 y.	♂ + ♀	(R)	No
van Selms et al.	-	-	-	-	No

An Asian research team also confirmed the thesis of muscular strengthening through exercises: in a questionnaire study, singers performed six times more muscle strengthening exercises than instrumentalists, with only half of the singers having complaints of orofacial pain, which often results from TMDs [39]. On the other hand, instrumentalists, for example violin or wind players, had a proven increased prevalence of TMDs due to other physical demands compared to singers [31, 32, 34]. In addition to the physical aspect, the fact that choral activity is often a pleasant and stress-reducing activity also plays a decisive role [37, 38].

Only one study from Sweden considered professional and recreational singers separately. The authors found that professional singers were both physically fitter but also more emotionally stressed than recreational singers [40].

In summary, according to current data, the question of the extent to which the qualitative and quantitative practice of singing influences the development of TMDs cannot be answered conclusively. To accurately assess the risk factors for singing, it is necessary to differentiate between professional and recreational singing compared to a control group and to eliminate the influencing factors of gender and age.

The aim of the present study is to investigate whether there are noticeable differences in self-reported TMDs symptoms between the three cohorts. The study is hypothesis generating. If tendencies can be identified here, further confirmatory studies should be conducted.

## Methods

The study reports recording to STROBE statement and checklist for cohort studies.

All human studies described were performed with the approval of the Ethics Committee Westfalia-Lippe and the University of Münster (2021-427-f-S) in accordance with national law and the Declaration of Helsinki.

### Female subjects

A total of 288 women participated in the descriptive cross-sectional study. All participants took part in the study voluntarily, were recruited at random and represented a quarter of the population with no affiliation to a dental speciality group. They did not receive any compensation for their participation. The study participants were divided into three cohorts (professional, recreational and nonsingers) of 96 subjects each. They were divided according to specific criteria, which were defined by the authors in advance of the study design. Recreational singers were defined as individuals who sang a maximum of five hours per week in their free time, while professional singers were defined as those who sang more than five hours per week. Subjects who did not sing were assigned to the nonsinger group (control group). This categorisation was made in consultation and discussion with professional singers, who were asked at what point they would consider someone to be a professional.

To put the influencing factors “age and gender” into perspective [18, 36, 41–44] and to create a homogeneous study population, only women aged 18 to 45 years were included in the study [18, 41]. Pregnant women as well as subjects with a legal representative or mental disability or patients who did not speak or write German were excluded. This took place indirectly through the prior viewing of the information sheet and the clearly formulated prohibition of participation under the latter conditions.

### Sample size calculation

The study aims to accurately estimate the probability of TMDs in each of the three cohorts. Point estimates, along with associated 95% Clopper-Pearson confidence intervals, are calculated for each cohort. Therefore the study’s sample size is determined using the normal distribution approximation, ensuring that the 95% confidence intervals do not exceed a maximum width of 10%, regardless of the unknown order of magnitude of π. If ${N}_{B}$, ${N}_{H}$, or ${N}_{N}$ represents the number of recruited professional singers, recreational singers, or nonsingers, respectively, the smallest upper bound for the width of the 95% confidence interval of π can be determined using φ to denote the distribution function of the standard normal distribution and α = 0.05, based on the normal distribution approximation.

1 $$\pm \frac{{\phi }^{-1}\left(1-\frac{\alpha }{2}\right)}{2\cdot \sqrt{{N}_{B}}} resp. \pm \frac{{\phi }^{-1}\left(1-\frac{\alpha }{2}\right)}{2\cdot \sqrt{{N}_{H}}} resp. \pm \frac{{\phi }^{-1}\left(1-\frac{\alpha }{2}\right)}{2\cdot \sqrt{{N}_{N}}}$$

Equating (1) with + 10% results in ${N}_{B}$ = ${N}_{H}$ = ${N}_{N}$ = 96. Therefore, the study aims to include 96 professional singers, 96 recreational singers, and 96 nonsingers, resulting in a total of 288 cases. This will ensure a maximum width of the 95% confidence intervals of π of 10% in each of the three cohorts.

### Questionnaire

The study was conducted using a paper-based self-assessment TMDs questionnaire modified for singers from the Department of Prosthodontics Münster. The first question about age ensured that only women between the ages of 18 and 45 were included in the study.

The questionnaire can be divided into different aspects: demographic data, qualitative and quantitative singing practice, previous dental treatments, TMDs-leading symptoms, localization of complaints, onset and times of complaints and accompanying symptoms.

Four questions were aimed at qualitative and quantitative singing practices. If a respondent denied any singing activity, she was classified as a nonsinger. If a respondent affirmed any singing activity, then she was counted as a singer. To classify the level of singing, the questions “How many days a week do you sing?” and “How many hours a day do you sing in total?” were asked. All those who sang for more than 5 h a week were classified as professional singers. Subjects who sang less than 5 h a week were classified as recreational singers. One question was aimed at other weekly physical activities for comparative reasons.

To identify possible TMDs sufferers, yes-no questions were asked about the following symptoms:

- pain in the temporomandibular joint and/or jaw.
- palpable or audible sounds (clicking or crepitus) in the temporomandibular joint during jaw movement.
- limitations and/or pain while opening, closing, chewing or lateral jaw movements.
- deviating mouth opening.
- restricted mouth opening.
- pain and/or hardening in the masticatory muscles around the temple, cheek or jaw angle.

Every respondent who answered at least three of the six leading symptoms in the affirmative was assigned to the group of TMDs sufferers. The diagnostic method is based on Ridder, which refers to data from the International Headache Society [45]. To measure if the mouth opening is restricted, the questionnaire included a tool: participants were asked to use an arrow printed on the questionnaire to find a point on their index and middle fingers corresponding to the length of the arrow (38 mm) and then check if the two fingers can be positioned between their incisors. If this is the case, it can be assumed that their mouth opening is not restricted. To assess whether the mouth opening was deviating, the patients were asked to open their mouths slowly several times in front of a mirror.

As soon as a leading symptom question has been answered in the affirmative, it should be specified since when and how severe (weekly or even more often) the complaints have been occurring. These and the following questions were not used for the statistical analysis of the aim of this study but will be used for further statistical analyses and are listed for the sake of completeness.

Questions were also asked about the status of dental care and previous treatments. The last four questions were aimed at the presence of the following accompanying symptoms or other complaints:

- pain and/or hardening in neck, shoulder, head or spine.
- the feeling that when biting down, individual teeth first touch before full contact can be made (nonsynchronized occlusion).
- current bruxism.
- bruxism in children.

In addition to the symptoms of TMDs, the questionnaire also included a question about occlusion. However, this did not serve to answer the study question but will be used for further analyses of accompanying symptoms.

### Data collection and study design

The data collection took place from July to December 2021. The completion of the questionnaire took on average ten minutes, and the initial reading required approximately five minutes. Before completing the questionnaire, the study participants were given time to read both the information sheet and the questionnaire and were able to ask questions before completion. The recruitment of nonsingers took place in public places. Recreational singers were recruited at resident choirs, theatres, karaoke bars, the college of music and the school for logopedics Muenster. While data collection from recreational and nonsingers was mainly performed in an analogue setting, the recruitment of professional singers was based on a digital compromise. With the help of social media, many professional singers were filtered and contacted via the “hashtags” they used, such as “#berufssängerin” or “#weddingsinger”. After the subjects had been informed and a thematic introduction had been given, the questionnaire was filled out and returned in anonymized form. The data were manually extracted from the TMDs questionnaires and transferred to a digital storage form (IBM SPSS Statistics 27).

### Data analysis

Using IBM SPSS Statistics 27, the working hypothesis that noticeable differences exist between the three cohorts regarding TMDs prevalence was statistically tested. The primary endpoint of the study was the occurrence of TMDs (binary: yes or no). For this purpose, all subjects who answered at least three leading symptom questions in the affirmative were marked as TMDs sufferers [45]. The presence of TMDs was thus the dependent variable and singing activity (“not singing at all”, “singing less than 5 h/week”, “singing more than 5 h/week”) was the independent variable. The primary objective of the study was to estimate the probability of TMDs with a given precision in each of these three cohorts. This was investigated by means of the Fisher-Freeman-Halton exact test (for 2xk contingency tables). All analyses were regarded as exploratory. Therefore, the term significant is intentionally replaced by noticeable in the context of this explorative study question. P values were displayed for descriptive reasons to study meaningful effects and were considered noticeable when *p* ≤ .05. For smaller values, Fisher’s exact test (for a 2 × 2 contingency table) was then used to test two cohorts in direct comparison. Using a binary logistic regression model, all variables that represent possible risk factors for the development of TMDs could then be exploratively tested.

## Results

Almost one in three (32.6%) of the study population suffered from TMDs. With a prevalence of 42% (95% CI = 31.9–52.5), professional singers were affected by TMDs more often than recreational singers (31%; 95% CI = 22–41.3) and almost twice as often as nonsingers (25%; 95% CI = 16.7–34.9). Using the Fisher-Freeman-Halton exact test, the differences in TMDs prevalence between the three groups were not shown to be noticeable (*p* = .053) but could be formulated as tendencies. Purely exploratively, two groups each were tested in a direct comparison using Fisher’s exact test. This revealed that professional singers had a higher TMDs prevalence than nonsingers (*p* = .021).

### Risk factors

Using a binary logistic regression model, age was exploratively tested for statistical conspicuity in TMDs sufferers and healthy participants in addition to professional/recreational singing. In this model, professional singing (*p* = .022; OR = 1.825; 95% CI = 1.092–3.050) could be considered a risk factor for the prevalence of TMDs. Using a second model (Table 2), all possible variables that could represent risk factors for the development of TMDs were tested in a purely exploratory manner. Of the thirteen variables, bruxism (*p* < .001; OR = 0.086; 95% CI = 0.036–0.203), nonsynchronized occlusion (*p* = .018; OR = 0.347; 95% CI = 0.144–0.833), pain and/or hardening in various areas (*p* = .013; OR = 0.287; 95% CI = 0.107–0.771), fillings, inlays, other restorations (*p* = .022; OR = 0.367; 95% CI = 0.156–0.864) and physical activity (*p* = .050; OR = 3.736; 95% CI = 0.999–13.971) were included in the model using the stepwise likelihood test (forward). Subjects with pain and/or hardening in the shoulder/neck/head/spine region who were not physically active, grind, or had fillings, inlays, other restorations, or a nonsynchronized occlusion were particularly affected by TMDs (Table 2).

**Table 2:** Within these five of thirteen criteria (physical activity, dental restorations, tension in various areas, nonsynchronized occlusion and bruxism), the three cohorts differed noticeably (*p* ≤ .05)

	Possible risk factors for TMDs			
	SD	*P*	OR	95% CI
Physical activity	0.673	***0.05***	3.736	0.999–13.971
Dental restorations	0.437	***0.022***	0.367	0.156–0.864
Tension in neck/shoulder/head/spine	0.504	***0.013***	0.287	0.107–0.771
Nonsynchronized occlusion	0.447	***0.018***	0.347	0.144–0.833
Bruxism	0.439	***0.000***	0.086	0.036–0.203

Since a higher TMDs prevalence of professional singers compared to nonsingers was found in the Fisher exact test, the second regression model was limited to professional and nonsingers for the explorative detection of risk factors for TMDs: professional singers and nonsingers differed in terms of TMDs prevalence only within two modalities: bruxism (*p* < .001; OR = 0.085; 95% CI = 0.038–0.188) and nonsynchronized occlusion (*p* = .013; OR = 0.332; 95% CI = 0.139–0.791).

### Demographic data

The study population could be considered homogeneous in terms of educational level and age. A total of 268 of 288 female subjects reported high school graduation (Abitur) as their highest degree. They did not differnoticeably within the three cohorts. Concerning the total study population, 91% reported being physically active. Nonsingers were more likely to report being physically active (97.9%) than professional singers (86.5%) (see Table 3). Fisher’s exact test showed that nonsingers were statistically more active than professional singers (*p* = .010).

**Table 3:** Frequency (%) of physical activity by professional, recreational and nonsingers

	Physical activity		
	Professional singers	Recreational singers	Nonsingers
Inactive	13.5	12.5	2.1
Up to 2 h per week	21.9	21.9	18.8
Up to 5 h per week	32.3	41.7	37.5
From 5 h per week	32.3	24	41.7

### Singing practice

**Fig. 1:**
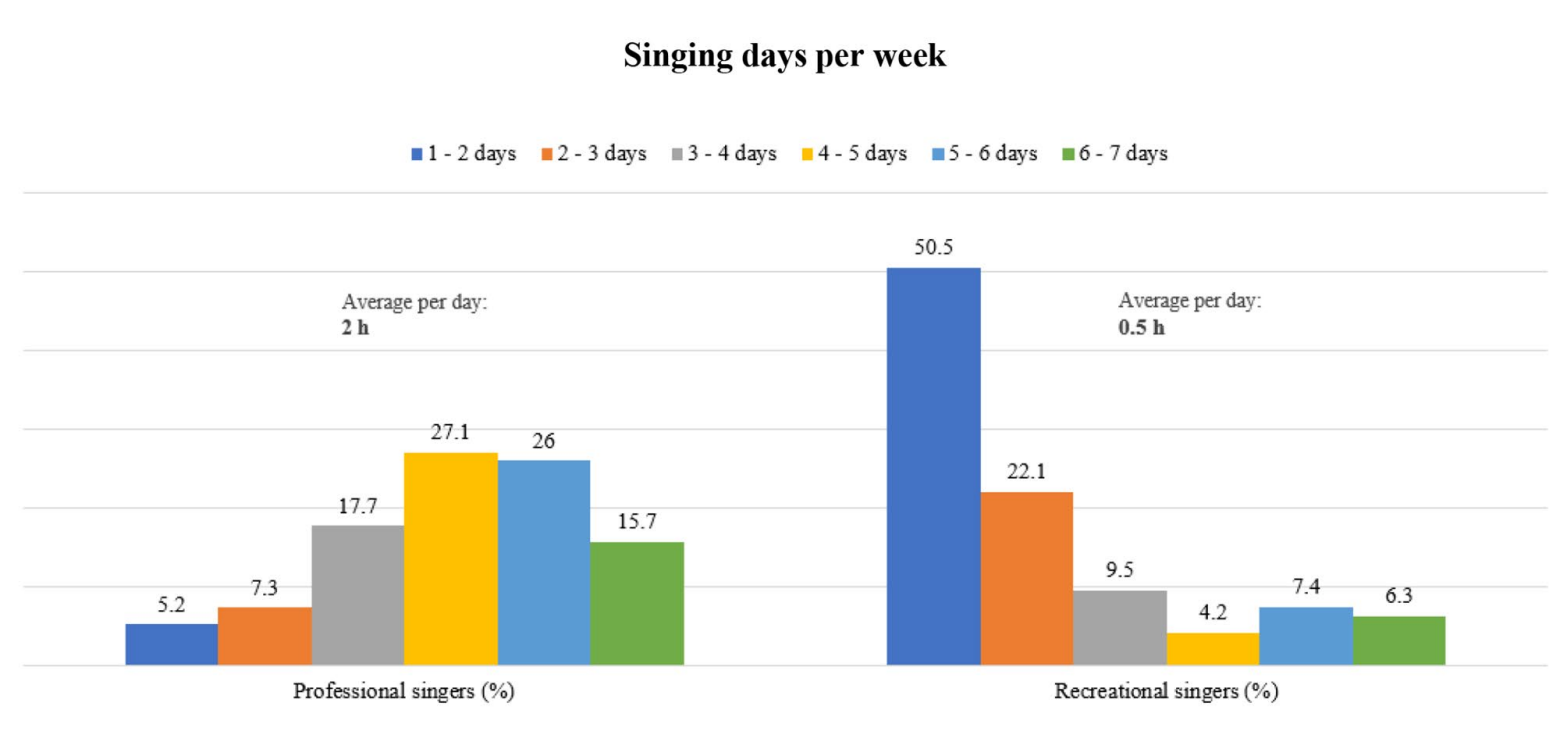
Quantitative singing load of professional and recreational singers

On average, recreational singers sang for twice as many years as professional singers. The daily and weekly singing load was higher among professional singers, as seen in Fig. 1.

### TMDs-leading symptoms

The most frequent leading symptoms within the three cohorts included noises in the temporomandibular joint and pain in the temporomandibular joint as well as in the masticatory muscles (Table 4). Comparing this with the distribution among TMDs sufferers, deviated mouth opening movement was reported most frequently (74.7%). This ranking did not differ noticeably among the three cohorts.

**Table 4:** Comparison of leading symptoms

	TMDs-leading symptoms	
	Number of cases	Frequency
Pain in the temporomandibular joint and/or jaw	97	33.7
Professional singers	36	37.5
Recreational singers	34	35.4
Nonsingers	27	28.1
Sounds in the temporomandibular joint	127	44.1
Professional singers	51	53.1
Recreational singers	44	45.8
Nonsingers	32	33.3
Limitations and/or pain during jaw movement	78	27.1
Professional singers	37	38.5
Recreational singers	23	24
Nonsingers	18	18.8
Deviating mouth opening	73	25.3
Professional singers	27	28.1
Recreational singers	31	32.3
Nonsingers	15	15.6
Restricted mouth opening	23	8
Professional singers	9	39.1
Recreational singers	10	43.5
Nonsingers	4	17.4
Pain and/or hardening in the masticatory muscles	98	34
Professional singers	42	43.8
Recreational singers	32	33.4
Nonsingers	24	25

The test statistics showed that professional singers tended to have a higher prevalence of TMDs than the control group and thus a stronger expression of the leading symptoms. Fisher’s exact test showed that the leading symptoms of jaw movement (*p* = .004), especially when opening the mouth, noises in the temporomandibular joint (*p* = .009) and pain and/or hardening in the muscles (*p* = .010), were noticeably more frequent in professional singers.

### Onset and times of complaints

Professional singers were affected by the leading symptoms of pain and noises in the temporomandibular joint several times a week, similar to nonsingers.

**Fig. 2:**
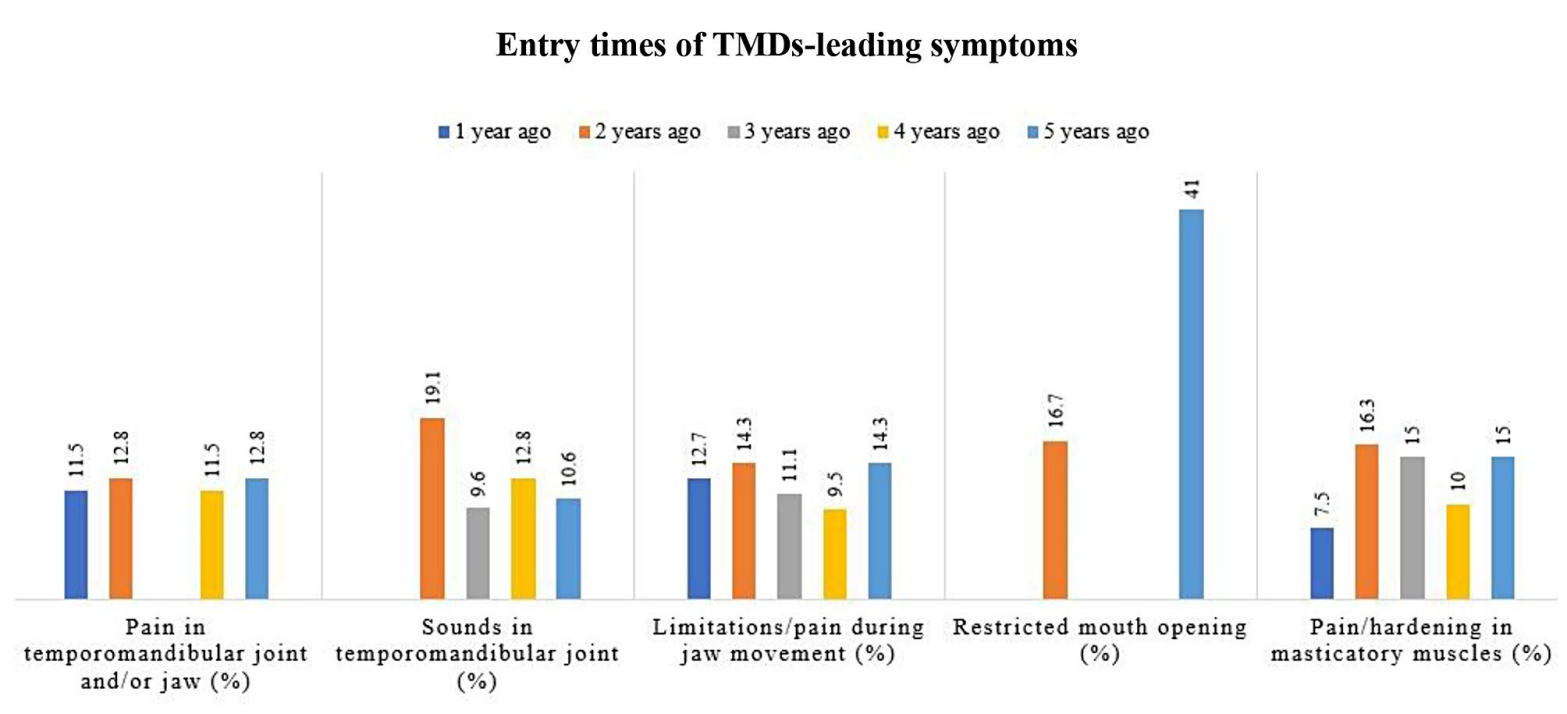
Comparison of the onset dates of the five leading TMDs leading within the entire study population

The main onset dates of the leading symptoms were between one and five years before the time of data collection. Within the three cohorts, the times of occurrence did not differnoticeably (Fig. 2).

### Previous treatments

One-quarter of the study population reported that they were currently wearing a bite splint and/or receiving logopedics or physiotherapeutic treatment. Across the three cohorts, the majority were currently receiving treatment, and professional singers were the most affected cohort.

### Accompanying symptoms

Two out of three subjects reported suffering from tension and/or pain in the neck, shoulder, head or spine region. There were no noticeable differences within the cohorts. TMDs patients had more complaints in almost all regions in an individual comparison with healthy study participants.

One in four subjects reported having nonsynchronized occlusion. While this finding was similar for professional and recreational singers, only half as many nonsingers were affected by this.

Professional singers, on the other hand, ground their teeth noticeably more often than the other two cohorts (*p* < .001). Every second professional singer suffered from bruxism. Recreational singers were affected 43.5% of the time and nonsingers 37.6%. One in five of the current gnashers also gnashed their teeth in childhood.

## Discussion

The aim of the present cross-sectional study is to check in an exploratory manner whether there are noticeable differences between the three cohorts (professional, recreational and nonsingers) regarding self-reported TMDs symptoms. The study is hypothesis generating.

Professional singers are more frequently affected by TMDs than recreational singers and nonsingers (*p* = .053). While this correlation of professional singers in comparison to recreational and nonsingers can be formulated as a statistical tendency, the exploratory direct comparison of the professionals to nonsingers is noticeable (*p* = .021). It could therefore be shown that recreational singing may have a slight influence and professional singing a greater influence on the development of TMDs. However, all p values should be interpreted as descriptive within the framework of the present exploratory study design and there are risks of bias behind the advantages of a questionnaire study. These must be critically discussed regarding the significance of the study results.

The results shown are compatible with the theory according to Franco and Andrus: a quantitatively high stress on the jaw through singing can lead to an overload of the masticatory apparatus [33]. In addition, a lack of recovery time can be seen as a predisposing factor for complaints in the area of the jaw and the voice-forming organs [29]. The authors refer to professional singers whose age and gender are not known. Other studies have been published only in part [29], refer indiscriminately to amateur and professional singers [40], examine only one cohort [33, 37, 38] or are systematic reviews [34, 35]. Including only the risk group, women aged 18 to 45 years [18, 41] have not yet been fulfilled by any study. This may be the reason why the results of previous studies in part or only slightly correspond to the results of the present study.

Every third female subject in the entire study population reported complaints in the sense of TMDs. Professional singers have a noticeably higher level of pain and discomfort than nonsingers on almost all leading symptoms. Abnormal jaw movement, noises in the temporomandibular joint and pain and/or hardening in the muscles are noticeably more common in professional singers than in nonsingers. In addition to the prevalence of TMDs, there are notable differences between professional, recreational and nonsingers regarding bruxism (compare Taddey [29]), occlusion, tension in various areas, dental care (fillings, inlays, other) and physical activity. In the total cohort, professional singers are most often gnashers (*p* < .001; OR = 0.086; 95% CI = 0.036–0.203), least regularly physically active (*p* = .050; OR = 3.736; 95% CI = 0.999–13.971), are provided with fillings, inlays, or others (*p* = .028; OR = 0.367; 95% CI = 0.156–0.864), have a nonsynchronized occlusion (*p* = .029; OR = 0.347; 95% CI = 0.144–0.833) and tension in the shoulder/neck/spine/head region (*p* = .025; OR = 0.287; 95% CI = 0.107–0.771). It is unclear whether these complaints are triggers for TMDs or must be interpreted as accompanying symptoms. If the latter were the case, there would be much to suggest that the increased prevalence of TMDs among professional singers can be explained by other causes, such as competitive/performance pressure, fear of failure/existence or stress. In addition, all recreational and nonsingers in the study population are employed, which may suggest that professional singing is a major psychosocial burden.

Another idea would be that the quantitatively high stress on the stomatognathic system (up to 9 h a day) leads to an increased risk of TMDs in professional singers. The emotional stress construct appears to represent a major risk for somatisation and TMDs [25, 26]. A small systematic review (12 included studies) sheds light on the association between work stress in employees and TMDs [46]. Every second study reported a positive correlation between work-related stress and TMDs, although the authors themselves consider the certainty of the evidence to be low due to the lack of eligible articles (four studies were finally included) and the methodological limitations. They also found the correlation that employees in the music industry have a frequent manifestation of sounds in the temporomandibular joint area. This is consistent with the results of the present study (Table 4), although it should be noted that it is unclear which job the subjects in the study by Aranha et al. actually do in the music industry. Other studies illuminate the biochemical and physiological level that the masticatory muscle activity measured by electromyography is higher in TMDs sufferers [47]. This can lead to an imbalance, muscular asymmetries and an overload of the temporomandibular joint and involved tissues. A Polish study provides evidence in favour of the theory that psychological stressors cause a different increase in masticatory muscle activity compared to control subjects [48]. Data available in the literature on TMDs prevalence in the general adult population range from 5 to 31% [5–7]. The data are based on self-assessment and clinical examination studies of women and men aged 18 to 75 years. The actual need for treatment is significantly lower at 16% [49]. The overall prevalence of the present study is above average at 33% and can be explained by the survey of the main risk group. Several studies report the prevalence of the main risk group to be 16 to 33% [50–52]. In addition, professional female singers (42%) clearly stand out as a risk group in comparison to the existing data.

The present study was designed to be exploratory, not confirmatory. Accordingly, values were obtained on the significance of individual correlations or differences. Therefore, values of p ≤ .050 were not interpreted as significant correlations or differences but as statistically noticeable since the type I error (rejecting the null hypothesis when it is actually true) was not controlled. So far, only limited data has been presented on this question, which is summarised in Table 1. Influencing factors such as age and gender were eliminated from the outset by the study design. Regarding other factors (level of education, regular dental visits, dental intervention in the sense of extraction of the third molars, singing genre and exercises), the three cohorts could be considered comparable. Concerning sporting activity, the difference was that nonsingers were statistically more active than professional singers (Table 3). This could result in a better body feeling and improved posture in nonsingers and positively influence their psychological well-being, depending on the type of sport and intensity. From this, the reverse conclusion could be drawn that the increased TMDs prevalence of professional singers was due to the low level of physical activity. A recent study showed that Pilates can significantly reduce anxiety, stress and TMDs (p ≤ .007) [53]. Regular physical activity is recommended as a therapeutic measure for bruxism [36]. Once this physical activity is performed professionally, the associated competition may be linked to stress for sufferers and thus may be a risk factor for TMDs [19, 27, 37]. The questionnaire design only considered the quantity of physical activity and not the quality (type of sport, club activity, intensity, etc.). This relationship should be considered and investigated in more detail in future studies. The following points can be discussed regarding the inclusion and categorisation of the study participants: It was not checked whether study participants who were pregnant, mentally handicapped, etc. had overlooked or even disregarded the exclusion criteria clearly formulated in the information sheet. There is therefore a certain risk that these participants took part in the study. However, the participation of all test subjects was without monetary incentive or other profit, so that the motivation of all persons who met the exclusion criteria can be considered low. The division of the cohorts into professional, recreational and nonsingers was based on the authors’ prior determination of the 5-hour per week limit (professional singers ≥ 5 h per week; recreational singers < 5 h per week). During the literature search for comparable studies, no clear published criteria for distinguishing between professional and amateur singers were found, so that no physiological or differentiated method can support this limit setting. The authors consulted professional singers in advance, most of whom believed anyone who sings at least one hour a day, five days a week, is no longer singing as a hobby. The average was therefore set at exactly 5 h per week. This can be reconciled with the intention of the exploratory study design, conceals a certain bias in every sense, but thus leaves room for further research. Even when age was narrowed down to the main risk group (18- to 45-year-olds), this range of 27 years may still be subject to large variances (Table 5) in terms of TMDs prevalence [21, 38, 40]. The subjects were randomly selected, and the age distribution was also random. Professional singers (median: 28 years) tended to be older than recreational singers (median: 22 years) and nonsingers (median: 23 years) in this study because they usually had to complete long vocal training. For example, in the group of 18- to 25-year-olds, there were twice as many recreational singers and nonsingers in comparison to professional singers (see Table 5). Through a binary logistic regression model, it could be shown that differences in age of the overall study population led to a different expression of TMDs; thus, age can be considered a risk factor for TMDs prevalence (p = .048; OR 1.047; 95% CI = 1.000-1.097). However, the difference between the cohorts (mean five and six years, respectively) is very subtle and does not represent a “generation gap”. Nevertheless, future studies should take this point into account.Through this study, it was shown that professional singers seem to be more likely to be affected by TMDs, which is usually very painful. It makes sense that those people using their jaw more in a professional way would be at higher risk of developing jaw related problems. However there are more factors to consider, such as competitive pressure, stress, anxiety/depression, and physical fitness, which require further investigation.

**Table 5:** TMDs prevalence divided into three different age groups

	TMDs among age groups	
	Number of cases	Frequency
18- to 25-year-olds (*n* = 184)	53	28.8
Professional singers (*n* = 36)	15	41.7
Recreational singers (*n* = 70)	17	24.3
Nonsingers (*n* = 78)	21	26.9
26- to 35-year-olds (*n* = 88)	36	40.9
Professional singers (*n* = 49)	23	46.9
Recreational singers (*n* = 23)	11	47.8
Nonsingers (*n* = 16)	2	12.5
36- to 45-year-olds (*n* = 16)	5	31.3
Professional singers (*n* = 11)	2	18.2
Recreational singers (*n* = 3)	2	66.7
Nonsingers (*n* = 2)	1	50

### Practical significance and limitations

The advantages of﻿ a questionnaire study always conceal risks of bias. The TMDs symptoms as well as the correct understanding of the research question were not clinically tested, and the diagnosis was based on the subjective self-assessment of all respondents. Without clinical validation, this method may be subject to discrepancies between findings and subjective well-being. This must be taken into consideration against the background of the results. However, it should be mentioned that the respondents remained anonymous and completed the survey voluntarily without remuneration, which would otherwise be expected to lead to more bias. It should also be considered that the study followed the overall aim of formulating trends in the cohorts and not clinically exact diagnoses. Nevertheless, professional singers stand out as a potential risk group that should not be neglected, which justifies the need for further research with the aim of raising awareness at an early stage and introducing preventive measures. Concrete therapy offers for those affected do not arise from the results of this study, as the concrete pathogenesis of the factors is still unclear. However, knowledge about risk factors is a first step towards preventing and treating the severity and duration of already manifested TMDs or avoiding recurrences.

Due to the multifactorial etiology of TMDs, the risk factors, specifically singing, are still quite unexplored. Further studies should be conducted to assess the psychological stress and the quality of physical activity of professional, recreational and nonsingers so that individual future therapy recommendations can be generated for affected people.

## Conclusions

Professional singers seem to be more often affected by TMDs than recreational singers and especially nonsingers. It remains to be investigated whether other influencing factors (stress, anxiety/depression, psychosocial factors such as pressure to perform/competition, state of health, quality of physical activity, etc.), which were not collected by the questionnaire, lead to the different accumulation of TMDs of affected (non)professional singers. The fact that every third test person was affected illustrates the importance of the clinical picture of TMDs in society. Above all, it emphasizes the desire of those affected to be able to speak, laugh, eat and sing again without pain, depression or even suicidal intentions.

## Data Availability

All data generated or analysed during this study are included in this published article.

## Abbreviations

TMDs: Temporomandibular disorders
resp.: Respectively
*N*_*B*_: Number of recruited professional singers
*N*_*H*_: Number of recruited recreational singers
*N*_*N*_: Number of recruited nonsingers
p: *p* value
OR: Odds ratio
CI: Confidence interval
i.e.: id est
etc.: et cetera

## References

[1] Schiffmann EL, Velly AM, Look JO, Hodges JS, Swift JQ, Decker KL (2014). Et. Al. Effects of four treatment strategies for temporomandibular joint closed lock. Int J Oral Maxillofac Surg.

[2] Ohrbach R, Dowkin SF (2016). The evolution of TMD diagnosis: past, Present, Future. J Dent Res.

[3] An JS, Jeon DM, Jung WS, Yang IH, Lim WH, Ahn SJ (2015). Influence of temporomandibular joint dic displacement on craniocervical posture and hyoid bone position. Am J Orthod Dentofac Orthop.

[4] Li DTS, Leung YY (2021). Temporomandibular disorders: current concepts and controversies in diagnosis and management. Diagnostics (Basel).

[5] Johansson A, Unell L, Carlsson GE, Söderfeldt B, Halling A (2003). Gender difference in symptoms related to temporomandibular disorders in a population of 50-year-old subjects. J Orofac Pain.

[6] de Kanter RJ, Truin GJ, Burgersdijk RC (1993). Prevalence in the Dutch adult population and a meta-analysis of signs and symptoms of temporomandibular disorder. J Dent Res.

[7] Valesan LF, Da-Cas CD, Reus JC, Denardin ACS, Garanhani RR, Bonotto D (2021). Prevalence of temporomandibular joint disorders: a systematic review and meta-analysis. Clin Oral Investig.

[8] Tran C, Ghahreman K, Huppa C, Gallagher JE (2022). Management of temporomandibular disorders: a rapid review of systematic reviews and guidelines. Int J Oral Maxillofac Surg.

[9] Paulino MR, Moreira VG, Lemos GA, da Silva PLP, Bonan PRF, Batista AUD (2018). Prevalence of signs and symptoms of temporomandibular disorders in college preparatory students: associations with emotional factors, parafunctional habits, and impact on quality of life. Cien Saude Colet.

[10] de Resende CMBM, da Silva Rocha LGD, de Paiva RP, da Silva Calcanti C, de Almeida EO, Roncalli AG (2020). Relationship between anxiety, quality of life, and sociodemographic characteristics and temporomandibular disorder. Oral Surg Oral Med Oral Pathol Oral Radiol.

[11] Bal B, Sarak G, Oral K (2022). Oral health-related quality of life and psychological states of dental students with temporomandibular disorders. J Dent Educ.

[12] Qamar Z, Alghamdi AMS, Haydarah NKB, Balateef AA, Alamoudi AA, Abumismar MA (2023). Impact of temporomandibular disorders on oral health-related quality of life: a systematic review and meta-analysis. J Oral Rehabil.

[13] Roithmann CC, da Silva CAG, Pattussi MP, Grossi ML (2021). Subjective sleep quality and temporomandibular disorders: systematic literature review and meta-analysis. J Oral Rehabil.

[14] Oliveira-Souza AISD, Sales LRDV, Coutinho ADDF, Armijo Olivo S, de Oliveira DA (2023). Oral health quality of life is associated to jaw function and depression in patients with myogenous temporomandibular dysfunction. Cranio.

[15] Park S, Heo HA, Yun KI, Pyo SW (2022). High prevalence of stress and suicidal ideation in women with temporomandibular disorder: a population-based cross-sectional survey. Cranio.

[16] Kalladka M, Young A, Thomas D, Heir GM, Quek SYP, Khan J (2022). The relation of temporomandibular disorders and dental occlusion: a narrative review. Quintessence Int.

[17] Thomas DC, Singer SR, Markman S (2023). Temporomandibular disorders and Dental occlusion: what do we know so far?. Dent Clin North Am.

[18] Michelotti A, Iodice G (2010). The role of orthodontics in temporomandibular disorders. J Oral Rehabil.

[19] Starr CL, McGrew C (2023). TMJ disorders in athletes. Curr Sports Med Rep.

[20] Karaman A, Sapan Z (2023). Evaluation of temporomandibular disorders, quality of life, and oral habits among dentistry students. Cranio.

[21] Glaros AG, Marszalek JM, Williams KB (2016). Longitudinal multilevel modeling of facial pain, muscle tension, and stress. J Dent Res.

[22] Reissmann DR, John MT, Schierz O, Seedorf H, Doering S (2012). Stress-related adaptive versus maladaptive coping and temporomandibular disorder pain. J Orofac Pain.

[23] Staniszewski K, Lygre H, Bifulco E et al. Temporomandibular disorders related to stress and HPA-Axis regulation. Pain Res Manag. 2018;1–7. 10.1155/2018/7020751 PMC5954859 29854038

[24] Wu G, Chen L, Fei H, Su Y, Zhu G, Chen Y (2013). Psychological stress may contribute to temporomandibular joint disorder in rats. J Surg Res.

[25] Yap AU, Sultana R, Natu VP (2022). Stress and emotional distress: their associations with somatic and temporomandibular disorder-related symptoms. Psychol Health Med.

[26] Vlăduțu D, Popescu SM, Mercuț R, Ionescu M, Scrieciu M, Glodeanu AD (2022). Associations between Bruxism, stress, and manifestations of Temporomandibular Disorder in Young Students. Int J Environ Res Public Health.

[27] Freiwald HC, Schwarzbach NP, Wolowski A (2021). Effects of competitive sports on temporomandibular dysfunction: a literature review. Clin Oral Invest.

[28] Deutscher Musikrat & Deutsches Musikinformationszentrum. Amateurmusizieren in Deutschland. Ergebnisse einer Repräsentativbefragung in der Bevölkerung ab 6 Jahre. https://miz.org/de/statistiken/amateurmusizieren-in-deutschland?term=amateurmusizieren%20statistik&position=3. Accessed 9 Feb 2024.

[29] Taddey JJ (1992). Musicians and temporomandibular disorders: prevalence and occupational etiologic considerations. Cranio.

[30] Attallah MM, Visscher CM, van Selms MKA, Lobbezoo F (2014). Is there an association between temporomandibular disorders and playing a musical instrument? A review of literature. J Oral Rehabil.

[31] Campos LGN, Pedrosa BH, Cavalcanti RVA (2021). Prevalence of temporomandibular disorders in musicians: a systematic review and meta-analysis. J Oral Rehabil.

[32] Rodríguez-Lozano FJ, Sáez-Yuguero MR, Bermejo-Fenoll A (2011). Orofacial problems in musicians: a review of the literature. Med Probl Perform Artist.

[33] Franco RA, Andrus JG (2007). Common diagnoses and treatments in professional voice users. Otolaryngol Clin N Am.

[34] van Selms MKA, Ahlberg J, Lobbezoo F, Visscher CM (2017). Evidence-based review on temporomandibular disorders among musicians. Occup Med.

[35] van Selms MKA, Wiegers JW, de Vries MW, Lobbezoo F, Visscher CM (2019). Zingen gaat niet gepaard met kaakklachten. Ned Tijdschr Tandheelkd.

[36] Chang TH, Da Yuh Y, Wu YT (2015). The association between temporomandibular disorders and joint hypermobility syndrome: a nationwide population-based study. Clin Oral Investig.

[37] Vaiano T, Guerrieri AC, Behlau M (2013). Body pain in classical choral singers. CoDAS.

[38] Caetano de Souza KA, Ferreira IMF, Mariotto LGS, Vidal CL, Neufeld CB, dos Reis AC (2019). Choir singing as an activity to manage anxiety and temporomandibular disorders: reports from a Brazilian sample. Psychol Music.

[39] Nair R, Tanikawa C, Ferreira JN (2023). Orofacial pain, musical performance and associated coping behaviors, psychological distress and disability among Asian young adults. J Clin Med.

[40] Grape C, Sandgren M, Hansson L-O, Ericson M, Theorell T (2003). Does singing promote well-being? An empirical study of professional and amateur singers during a singing lesson. Integr Physiol Behav Sci: off J Pavlov Soc.

[41] Koidis PT, Zarifi A, Grigoriadou E, Garefis P (1993). Effect of age and sex on craniomandibular disorders. J Prosthet Dent.

[42] Michelotti A, Rongo R, D’Antò V, Bucci R (2020). Occlusion, orthodontics, and temporomandibular disorders: cutting edge of the current evidence. J World Fed Orthod.

[43] Wänman A (1987). Craniomandibular disorders in adolescents. A longitudinal study in an urban Swedish population. Swed Dent J.

[44] Bueno CH, Pereira DD, Pattussi MP, Grossi PK, Grossi ML (2018). Gender differences in temporomandibular disorders in adult populational studies: a systematic review and meta-analysis. J Oral Rehabil.

[45] Ridder PH (2019). Craniomandibular Dysfunction (4th). What is craniomandibular dysfunction? [in German].

[46] Aranha RL, de Martins B, de Aguilar R, Moreno-Drada DR, Sohn JA, de Martins W (2021). C, u. a. association between stress at work and Temporomandibular disorders: a systematic review. Biomed Res Int.

[47] Janal MN, Lobbezoo F, Quigley KS, Raphael KG (2021). Stress-evoked muscle activity in women with and without chronic myofascial face pain. J Oral Rehabil.

[48] Zieliński G, Ginszt M, Zawadka M, Rutkowska K, Podstawka Z, Szkutnik J (2021). u. a. The relationship between stress and masticatory muscle activity in female students. J Clin Med.

[49] Al-Jundi MA, John MT, Setz JM, Szentpétery A, Kuss O (2008). Meta-analysis of treatment need for temporomandibular disorders in adult nonpatients. J Orofac Pain.

[50] Lövgren A, Österlund C, Ilgunas A, Lampa E, Hellström F (2018). A high prevalence of TMD is related to somatic awareness and pain intensity among healthy dental students. Acta Odontol Scand.

[51] Sójka A, Stelcer B, Roy M, Mojs E, Pryliński M. Is there a relationship between psychological factors and TMD? Brain Behav. 2019;9. 10.1002/brb3.1360 PMC7649956 31339236

[52] Heß S. Zusammenhänge zwischen kraniomandibulärer Dysfunktion und pathologischen Veränderungen des Bewegungsapparates im Patientengut einer allgemeinzahnärztlichen Praxis. https://nbn-resolving.org/urn:nbn:de:gbv:9-000961-4. Accessed 9 Feb 2024.

[53] Fleming KM, Campbell M, Herring MP (2020). Acute effects of Pilates on mood states among young adult males. Complement Ther Med.

